# Why optional stopping can be a problem for Bayesians

**DOI:** 10.3758/s13423-020-01803-x

**Published:** 2020-11-18

**Authors:** Rianne de Heide, Peter D. Grünwald

**Affiliations:** 1 Leiden University, Leiden, Amsterdam The Netherlands; 2 The Netherlands Centre for Mathematics & Computer Science (CWI), Amsterdam, The Netherlands

**Keywords:** Bayesian statistics, Hypothesis testing, Model selection, Statistical inference

## Abstract

Recently, optional stopping has been a subject of debate in the Bayesian psychology community. Rouder (*Psychonomic Bulletin & Review* *21*(2), 301–308, 2014) argues that optional stopping is no problem for Bayesians, and even recommends the use of optional stopping in practice, as do (Wagenmakers, Wetzels, Borsboom, van der Maas & Kievit, *Perspectives on Psychological Science* *7*, 627–633, 2012). This article addresses the question of whether optional stopping is problematic for Bayesian methods, and specifies under which circumstances and in which sense it is and is not. By slightly varying and extending Rouder’s (*Psychonomic Bulletin & Review* *21*(2), 301–308, 2014) experiments, we illustrate that, as soon as the parameters of interest are equipped with default or pragmatic priors—which means, in most practical applications of Bayes factor hypothesis testing—resilience to optional stopping can break down. We distinguish between three types of default priors, each having their own specific issues with optional stopping, ranging from no-problem-at-all (type 0 priors) to quite severe (type II priors).

## Introduction

*P* value-based null-hypothesis significance testing (NHST) is widely used in the life and behavioral sciences, even though the use of *p* values has been severely criticized for at least the last 50 years. During the last decade, within the field of psychology, several authors have advocated the Bayes factor as the most principled alternative to resolve the problems with *p* values. Subsequently, these authors have made an admirable effort to provide practitioners with *default Bayes factors* for common hypothesis tests. Key references include, among many others, Rouder, Speckman, Sun, Morey, and Iverson (2009), Jamil, Ly, Morey, Love, Marsman, and Wagenmakers (2016), Rouder, Morey, Speckman, and Province (2012).

We agree with the objections against the use of *p* value-based NHST and the view that this paradigm is inappropriate (or at least far from optimal) for scientific research, and we agree that the Bayes factor has many advantages. However, as also noted by Gigerenzer and Marewski (2014), it is not the panacea for hypothesis testing that a lot of articles make it appear. The Bayes factor has its limitations (cf. also Tendeiro & Kiers, 2019), and it seems that the subtleties of when those limitations apply sometimes get lost in the overwhelming effort to provide a solution to the pervasive problems of *p* values.

In this article, we elucidate the intricacies of handling optional stopping with Bayes factors, primarily in response to Rouder (2014). *Optional stopping* refers to ‘looking at the results so far to decide whether or not to gather more data’, and it is a desirable property of a hypothesis test to be able to *handle optional stopping*. The key question is whether Bayes factors can or cannot handle optional stopping. Yu, Sprenger, Thomas, and Dougherty (2014), Sanborn and Hills (2014) and Rouder (2014) tried to answer this question from different perspectives and with different interpretations of the notion of handling optional stopping. Rouder (2014) illustrates, using computer simulations, that optional stopping is not a problem for Bayesians, also citing Lindley (1957) and Edwards, Lindman, and Savage (1963) who provide mathematical results to a similar (but not exactly the same) effect. Rouder used the simulations to concretely illustrate more abstract mathematical theorems; these theorems are indeed formally proven by Deng, Lu, and Chen (2016) and, in a more general setting, by Hendriksen, De Heide, and Grünwald (2018). Other early work indicating that optional stopping is not a problem for Bayesians includes Savage (1972) and Good (1991). We briefly return to all of these in Section Other conceptualizations of optional stopping.

All this earlier work notwithstanding, we maintain that optional stopping can be a problem for Bayesians—at least for *pragmatic Bayesians* who are either willing to use so-called ‘default’, or ‘convenience’ priors, or otherwise are willing to admit that their priors are imperfect and are willing to subject them to robustness analyses. In practice, nearly all statisticians who use Bayesian methods are ‘pragmatic’ in this sense.

Rouder (2014) was written mainly in response to Yu et al., (2014), and his main goal was to show that Bayesian procedures retain a clear interpretation under optional stopping. He presents a criterion which, if it holds for a given Bayesian method, indicates that, in some specific sense, it performs as one would hope under optional stopping. The main content of this article is to investigate this criterion, which one may call *prior-based calibration*, for common testing scenarios involving default priors. We shall encounter two types of default priors, and we shall see that Rouder’s calibration criterion—while indeed providing a clear *interpretation* to Bayesian optional stopping whenever defined—is in many cases either of limited *relevance* (type I priors) or *undefined* (type II priors).

We consider a strengthening of Rouder’s check which we call *strong calibration*, and which remains meaningful for all default priors. Then, however, we shall see that strong calibration fails to hold under optional stopping for all default priors except, interestingly, for a special type of priors (which we call “type 0 priors”) on a special (but common) type of nuisance parameters. Since these are rarely the only parameters incurring in one’s models, one has to conclude that optional stopping is usually a problem for pragmatic Bayesians—at least under Rouder’s calibration criterion of handling optional stopping. There exist (at least) two other reasonable definitions of ‘handling optional stopping’, which we provide in Section Other conceptualizations of optional stopping. There we also discuss how, under these alternative definitions, type I priors are sometimes less problematic, but type II priors still are. As explained in the conclusion (Section Discussion and conclusion, the overall crux is that default and pragmatic priors represent *tools* for inference just as much or even more than *beliefs* about the world, and should thus be equipped with a precise prescription as to what type of inferences they can and cannot be used for. A first step towards implementing this radical idea is given by one of us in the recent paper *Safe Probability* (Grünwald, 2018).

Readers who are familiar with Bayesian theory will not be too surprised by our conclusions: It is well known that what we call type II priors violate the *likelihood principle* (Berger and Wolpert, 1988) and/or lead to (mild) forms of *incoherence* (Seidenfeld, 1979) and, because of the close connection between these two concepts and optional stopping, it should not be too surprising that issues arise. Yet it is still useful to show how these issues pan out in simple computer simulations, especially given the apparently common belief that optional stopping is *never* a problem for Bayesians. The simulations will also serve to illustrate the difference between the subjective, pragmatic and objective views of Bayesian inference, a distinction which matters a lot and which, we feel, has been underemphasized in the psychology literature—our simulations may in fact serve to help the reader decide what viewpoint he or she likes best.

In Section Bayesian probability and Bayes factors we explain important concepts of Bayesianism and Bayes factors. Section Handling optional stopping in the calibration sense explains Rouder’s calibration criterion and repeats and extends Rouder’s illustrative experiments, showing the sense in which optional stopping is indeed not a problem for Bayesians. Section When problems arise: Subjective vs. pragmatic and default priors then contains additional simulations indicating the problems with default priors as summarized above. In Section Other conceptualizations of optional stopping we discuss conceptualizations of ‘handling optional stopping’ that are different from Rouder’s; this includes an explication of the purely subjective Bayesian viewpoint as well as an explication of a frequentist treatment of handling optional stopping, which only concerns sampling under the null hypothesis. We illustrate that some (not all!) Bayes factor methods can handle optional stopping in this frequentist sense. We conclude with a discussion of our findings in Section Discussion and conclusion.

## Bayesian probability and Bayes factors

Bayesianism is about a certain interpretation of the concept *probability*: as *degrees of belief*. Wagenmakers (2007) and Rouder (2014) give an intuitive explanation for the different views of frequentists and Bayesians in statistics, on the basis of coin flips. The frequentists interpret probability as a limiting frequency. Suppose we flip a coin many times, if the probability of heads is 3/4, we see a proportion of 3/4 of all those coin flips with heads up. Bayesians interpret probability as a degree of belief. If an agent believes the probability of heads is 3/4, she believes that it will be three times more likely that the next coin flip will result in heads than tails; we return to the operational meaning of such a ‘belief’ in terms of betting in Section Other conceptualizations of optional stopping.

A Bayesian first expresses this belief as a probability function. In our coin-flipping example, it might be that the agent believes that it is more likely that the coin is biased towards heads, which the probability function thus reflects. We call this the *prior distribution*, and we denote^1^ it by $\mathbb {P}(\theta )$, where *𝜃* is the parameter (or several parameters) of the model. In our example, *𝜃* expresses the bias of the coin, and is a real number between 0 and 1. After the specification of the prior, we conduct the experiment and obtain the data *D* and the likelihood $\mathbb {P}(D | \theta )$. Now we can compute the *posterior distribution* $\mathbb {P}(\theta | D)$ with the help of *Bayes’ theorem*:

1 $$ \begin{array}{@{}rcl@{}} \mathbb{P}(\theta | D) = \frac{\mathbb{P}(D | \theta)\mathbb{P}(\theta)}{\mathbb{P}(D)}. \end{array} $$

Rouder (2014) and Wagenmakers (2007) provide a clear explanation of Bayesian hypothesis testing with Bayes factors (Jeffreys, 1961; Kass and Raftery, 1995), which we repeat here for completeness. Suppose we want to test a null hypothesis ${\mathscr{H}}_{0}$ against an alternative hypothesis ${\mathscr{H}}_{1}$. A hypothesis can consist of a single distribution, for example: ‘the coin is fair’. We call this a *simple hypothesis*. A hypothesis can also consist of two or more, or even infinitely many hypotheses, which we call a *composite hypothesis*. An example is: ‘the coin is biased towards heads’, so the probability of heads can be any number between 0.5 and 1, and there are infinitely many of those numbers. Suppose again that we want to test ${\mathscr{H}}_{0}$ against ${\mathscr{H}}_{1}$. We start with the so-called *prior odds*: $\mathbb {P}({\mathscr{H}}_{1}) / \mathbb {P}({\mathscr{H}}_{0})$, our belief before seeing the data. Let’s say we believe that both hypotheses are equally probable, then our prior odds are 1-to-1. Next we gather data *D*, and update our odds with the new knowledge, using Bayes’ theorem (Eq. 1):

2 $$ \begin{array}{@{}rcl@{}} {\texttt{post-odds}(\mathcal{H}_{1} \text{vs.} \mathcal{H}_{0}  | D)} = \frac{\mathbb{P}(\mathcal{H}_{1} | D)}{\mathbb{P}(\mathcal{H}_{0} | D)} = \frac{\mathbb{P}(\mathcal{H}_{1})}{\mathbb{P}(\mathcal{H}_{0})} \frac{\mathbb{P}(D | \mathcal{H}_{1})}{\mathbb{P}(D | \mathcal{H}_{0})}. \end{array} $$

The left term is called *posterior odds*, it is our updated belief about which hypothesis is more likely. Right of the prior odds, we see the *Bayes factor*, the term that describes how the beliefs (prior odds) are updated via the data. If we have no preference for one hypothesis and set the prior odds to 1-to-1, we see that the posterior odds are just the Bayes factor. If we test a composite ${\mathscr{H}}_{0}$ against a composite ${\mathscr{H}}_{1}$, the Bayes factor is a ratio of two likelihoods in which we have two or more possible values of our parameter *𝜃*. Bayesian inference tells us how to calculate $\mathbb {P}(D \mid {\mathscr{H}}_{j})$: we integrate out the parameter with help of a prior distribution $\mathbb {P}(\theta )$, and we write Eq. 2 as:

3 $$ \begin{array}{@{}rcl@{}} {\texttt{post-odds}(\mathcal{H}_{1} \text{vs.} \mathcal{H}_{0}  | D)} = \frac{\mathbb{P}(\mathcal{H}_{1} | D)}{\mathbb{P}(\mathcal{H}_{0} | D)} = \frac{\mathbb{P}(\mathcal{H}_{1})}{\mathbb{P}(\mathcal{H}_{0})} \frac{{\int}_{\theta_{1}} \mathbb{P}(D | \theta_{1}) \mathbb{P} (\theta_{1}) \mathrm{d} \theta_{1}}{{\int}_{\theta_{0}} \mathbb{P}(D | \theta_{0}) \mathbb{P} (\theta_{0}) \mathrm{d} \theta_{0}} \end{array} $$

where *𝜃*_0_ denotes the parameter of the null hypothesis ${\mathscr{H}}_{0}$, and similarly, *𝜃*_1_ is the parameter of the alternative hypothesis ${\mathscr{H}}_{1}$. If we observe a Bayes factor of 10, it means that the *change* in odds from prior to posterior in favor of the alternative hypothesis ${\mathscr{H}}_{1}$ is a factor 10. Intuitively, the Bayes factor provides a measure of whether the data have increased or decreased the odds on ${\mathscr{H}}_{1}$ relative to ${\mathscr{H}}_{0}$.

## Handling optional stopping in the calibration sense

Validity under optional stopping is a desirable property of hypothesis testing: we gather some data, look at the results, and decide whether we stop or gather some additional data. Informally we call ‘peeking at the results to decide whether to collect more data’ *optional stopping*, but if we want to make more precise what it means if we say that a test can handle optional stopping, it turns out that different approaches (frequentist, subjective Bayesian and objective Bayesian) lead to different interpretations or definitions. In this section we adopt the definition of handling optional stopping that was used by Rouder, and show, by repeating and extending Rouder’s original simulation, that Bayesian methods do handle optional stopping in this sense. In the next section, we shall then see that for ‘default’ and ‘pragmatic’ priors used in practice, Rouder’s original definition may not always be appropriate—indicating there are problems with optional stopping after all.

### Example 0: Rouder’s example

We start by repeating Rouder’s (2014) second example, so as to explain his ideas and re-state his results. Suppose a researcher wants to test the null hypothesis ${\mathscr{H}}_{0}$ that the mean of a normal distribution is equal to 0, against the alternative hypothesis ${\mathscr{H}}_{1}$ that the mean is not 0: we are really testing whether *μ* = 0 or not. In Bayesian statistics, the composite alternative ${\mathscr{H}}_{1}: \mu \neq 0$ is incomplete without specifying a prior on *μ*; like in Rouder’s example, we take the prior on the mean to be a standard normal, which is a fairly standard (though by no means the only common) choice (Berger, 1985; Bernardo & Smith, 1994). This expresses a belief that small effect sizes are possible (though the prior probability of the mean being *exactly* 0 is 0), while a mean as large as 1.0 is neither typical nor exceedingly rare. We take the variance to be 1, such that the mean equals the effect size. We set our prior odds to 1-to-1: This expresses a priori indifference between the hypotheses, or a belief that both hypotheses are really equally probable. To give a first example, suppose we observe *n* = 10 observations Now we can observe the data and update our prior beliefs. We calculate the posterior odds, in our case equal to the Bayes factor, via Eq. 2 for data *D* = (*x*_1_,…,*x*_*n*_):

4 $$ \begin{array}{@{}rcl@{}} {\text{\texttt{post-odds}}( \mathcal{H}_1 \/\text{vs.}\/ \mathcal{H}_{0}  | {x_{1},\ldots,x_{n}})} &= \frac{1}{1} \cdot \frac{\exp\left\lbrace \frac{n^{2} \overline{x}^{2}}{2(n+1)} \right\rbrace}{\sqrt{n+1}} \end{array} $$

where *n* is the sample size (ten in our case), and $\overline {x}$ is the sample mean. Suppose we observe posterior odds of 3.5-to-1 in favor of the null.

#### Calibration, mathematically

As Rouder writes: ‘If a replicate experiment yielded a posterior odds of 3.5-to-1 in favor of the null, then we expect that the null was 3.5 times as probable as the alternative to have produced the data.’ In mathematical language, this can be expressed as

5 $$ {\text{\texttt{post-odds}}( \mathcal{H}_1 \/\text{vs.}\/ \mathcal{H}_{0}  | {\text{``\texttt{post-odds}}( \mathcal{H}_{1} \/\text{vs.}\/\mathcal{H}_{0} |  }{x_{1},\ldots, x_{n}})} = a") \ =\ a, $$

for the specific case *n* = 10 and *a* = 1/3.5; of course we would expect this to hold for general *n* and *a*. The quotation marks indicate that we condition on an event, i.e. a set of different data realizations; in our case this is the set of all data *x*_1_,…,*x*_*n*_ for which the posterior odds are *a*. We say that Eq. 5 expresses *calibration of the posterior odds*. To explain further, we draw the analogy to weather forecasting: consider a weather forecaster who, on each day, announces the probability that it will rain the next day at a certain location. It is standard terminology to call such a weather forecaster *calibrated* if, on average on those days for which he predicts ‘probability of rain is 30*%*’, it rains about 30*%* of the time, on those days for which he predicts 40*%*, it rains 40*%* of the time, and so on. Thus, although his predictions presumably depend on a lot of data such as temperature, air pressure at various locations etc., given *only* the fact that this data was such that he predicts *a*, the actual probability is *a*. Similarly, given only the fact the posterior odds based on the full data are *a* (but not given the full data itself), the posterior odds should still be *a* (readers who find Eq. 5 hard to interpret are urged to study the simulations below).

Indeed, it turns out that Eq. 5 is the case. This can be shown either as a mathematical theorem, or, as Rouder does, by computer simulation. At this point, the result is merely a sanity check, telling us that Bayesian updating is not crazy, and is not really surprising. Now, instead of a fixed *n*, let us consider optional stopping: we keep adding data points until the posterior odds are at least 10-to-1 for either hypothesis, unless a maximum of 25 data points was reached. Let *τ* be the sample size (which is now data-dependent) at which we stop; note that *τ* ≤ 25. Remarkably, it turns out that we still have

6 $$ {\text{\texttt{post-odds}}( \mathcal{H}_1 \/\text{vs.}\/ \mathcal{H}_{0}  | {\text{``\texttt{post-odds}}(\mathcal{H}_{1} \/\text{vs.}\/\mathcal{H}_{0} |  }{x_{1},\ldots, x_{\tau}})} = a") \ =\ a, $$

for this (and in fact any other data-dependent) stopping time *τ*. In words, *the posterior odds remain calibrated under optional stopping*. Again, this can be shown formally, as a mathematical theorem (we do so in Hendriksen et al., (2018); see also Deng et al., (2016)).

#### Calibration, proof by simulation

Following (Yu et al., 2014) and Sanborn and Hills (2014), Rouder uses computer simulations, rather than mathematical derivation, to elucidate the properties of analytic methods. In Rouder’s words ‘this choice is wise for a readership of experimental psychologists. Simulation results have a tangible, experimental feel; moreover, if something is true mathematically, we should be able to see it in simulation as well’. Rouder illustrates both Eqs. 5 and 6 by a simulation which we now describe.

**Fig. 1:**
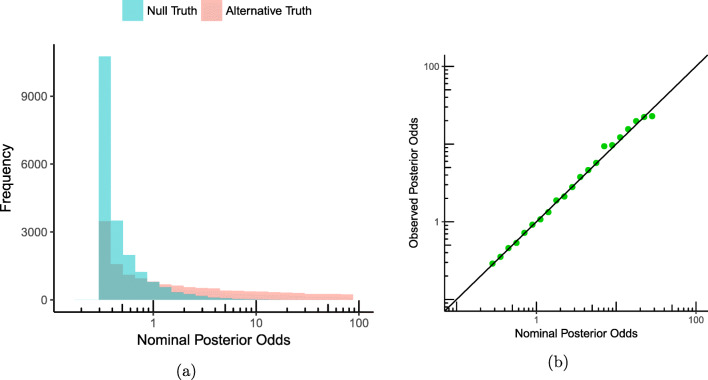
The interpretation of the posterior odds in Rouder’s experiment, from 20,000 replicate experiments. **a** The empirical sampling distribution of the posterior odds as a histogram under ${\mathscr{H}}_{0}$ and ${\mathscr{H}}_{1}$. **b** Calibration plot: the observed posterior odds as a function of the nominal posterior odds

Again, we draw data from the null hypothesis: say *n* = 10 observations from a normal distribution with mean 0 and variance 1, but now we repeat this procedure 20,000 times, and we see the distribution of the posterior odds plotted as the blue histogram on the log scale in Fig. 1a. We also sample data from the alternative distribution ${\mathscr{H}}_{1}$: first we sample a mean from a standard normal distribution (readers that consider this ‘sampling from the prior’ to be strange are urged to read on), and then we sample ten observations from a normal distribution with this just obtained mean, and variance 1. Next, we calculate the posterior odds from Eq. 4. Again, we perform 20,000 replicate experiments of ten data points each, and we obtain the pink histogram in Fig 1a. We see that for the null hypothesis, most samples favor the null (the values of the Bayes factor are smaller than 1), for the alternative hypothesis we see that the bins for higher values of the posterior odds are higher.

In terms of this simulation, Rouder’s claim that, ‘If a replicate experiment yielded a posterior odds of 3.5-to-1 in favor of the null, then we expect that the null was 3.5 times as probable as the alternative to have produced the data’, as formalized by Eq. 5, now says the following: if we look at a specific bin of the histogram, say at 3.5, i.e., the number of all the replicate experiments that yielded approximately a posterior odds of 3.5, then the bin from ${\mathscr{H}}_{1}$ should be about 3.5 times as high as the bin from ${\mathscr{H}}_{0}$. Rouder calls the ratio of the two histograms the *observed posterior odds*: the ratio of the binned posterior odds counts we observe from the simulation experiments we did. What we expect the ratio to be for a certain value of the posterior odds is what he calls the *nominal posterior odds*. We can plot the observed posterior odds as a function of the nominal posterior odds, and we see the result in Fig. 1b. The observed values agree closely with the nominal values: all points lie within simulation error on the identity line, which can be considered as a ‘proof of Eq. 5 by simulation’.

Rouder (2014) repeats this experiment under optional stopping: he ran a simulation experiment with exactly the same setup, except that in each of the 40,000 simulations, sampling occurred until the posterior odds were at least 10-to-1 for either hypothesis, unless a maximum of 25 observations was reached. This yielded a figure indistinguishable from Fig. 1b, from which Rouder concluded that ‘the interpretation of the posterior odds holds with optional stopping’; in our language, *the posterior odds remain calibrated under optional stopping*—it is a proof, by simulation, that Eq. 6 holds. From this and similar experiments, Rouder concluded that Bayes factors still have a clear interpretation under optional stopping (we agree with this for what we call below type 0 and I priors, not type II), leading to the claim/title ‘optional stopping is no problem for Bayesians’ (for which we only agree for type 0 and purely subjective priors).

#### Is sampling from the prior meaningful?

When presenting Rouder’s simulations to other researchers, a common concern is: ‘how can sampling a parameter from the prior in ${{\mathscr{H}}_{1}}$ be meaningful? In any real-life experiment, there is just one, fixed population value, i.e., one fixed value of the parameter that governs the data.’ This is indeed true, and not in contradiction with Bayesian ideas: Bayesian statisticians put a distribution on parameters in ${{\mathscr{H}}_{1}}$ that expresses their uncertainty about the parameter, and that should not be interpreted as something that is ‘sampled’ from. Nevertheless, Bayesian posterior odds calculations are done by calculating weighted averages via integrals, and the results are *mathematically equivalent* to what one gets if, as above, one samples a parameter from the prior, and the data from the parameter, and then takes averages over many repetitions. We (and Rouder) really want to establish Eqs. 5 and 6 (which can be interpreted without resorting to sampling a parameter from a prior), and we note that it is equivalent to the curve in Fig. 1b coinciding with the diagonal.

Some readers of an earlier draft of this paper concluded that, given its equivalence to an experiment involving sampling from the prior, which feels meaningless to them, Eq. 6 is itself invariably meaningless. Instead, they claim, because in real-life the parameter often has one specific fixed value, one should look at what happens under sampling under fixed parameter values. Below we shall see that if we look at such *strong calibration*, we sometimes (Example 1) still get calibration, but usually (Example 2) we do not; so such readers will likely agree with our conclusion that ‘optional stopping can be a problem for Bayesians’, even though they would disagree with us on some details, because we do think that Eq. 6 can be a meaningful statement for some, but not all priors. To us, the importance of the simulations is simply to verify Eq. 6 and, later on (Example 2), to show that Eq. 8, the stronger analogue of Eq. 6 that we would like to hold for default priors, does not always hold.

### Example 1: Rouder’s example with a nuisance parameter

We now adjust Rouder’s example to a case where we still want to test whether *μ* = 0, but the variance *σ*^2^ is unknown. Posterior calibration will still be obtained under optional stopping; the example mainly serves to gently introduce the notions of *improper prior* and *strong vs. prior calibration*, that will play a central role later on. So, ${{\mathscr{H}}_{0}}$ now expresses that the data are independently normally distributed with mean 0 and some unknown variance *σ*^2^, and ${{\mathscr{H}}_{1}}$ expresses that the data are normal with variance *σ*^2^, and some mean *μ*, where the uncertainty about *μ* is once again captured by a normal prior: the mean is distributed according to a normal with mean zero and variance (again) *σ*^2^ (this corresponds to a standard normal distribution on the effect size). If *σ*^2^ = 1, this reduces to Rouder’s example; but we now allow for arbitrary *σ*^2^. We call *σ*^2^ a *nuisance parameter*: a parameter that occurs in both models, is not directly of interest, but that needs to be accounted for in the analysis. The setup is analogous to the standard 1-sample frequentist *t* test, where we also want to test whether a mean is 0 or not, without knowing the variance; in the Bayesian approach, such a test only becomes defined once we have a prior for the parameters. For *μ* we choose a normal,^2^ for the nuisance parameter *σ* we will make the standard choice of Jeffreys’ prior for the variance: ${\mathbb P}_{\text {\textsc {j}}}(\sigma ) := 1/\sigma $ Rouder et al., (2009). To obtain the Bayes factor for this problem, we integrate out the parameter *σ* cf. Eq. 3. Again, we assign prior odds of 1-to-1, and obtain the posterior odds:

$$ \begin{array}{@{}rcl@{}} {\texttt{post-odds}(\mathcal{H}_{1} \text{vs.} \mathcal{H}_{0}  | D)} &= \frac{1}{1} \frac{ {\int}_{0}^{\infty} \frac{1}{\sigma} {\prod}_{i=1}^{n} \frac{1}{\sqrt{2\pi\sigma^{2}}} \exp\left( -\frac{{{x}_{i}^{2}}}{2\sigma^{2}} \right) \mathrm{d}\sigma }{{\int}_{0}^{\infty} \frac{1}{\sigma} {\int}_{-\infty}^{\infty} \frac{1}{\sqrt{2\pi\sigma^{2}}} \exp\left( -\frac{\mu}{2\sigma^{2}} \right) {\prod}_{i=1}^{n} \frac{1}{\sqrt{2\pi\sigma^{2}}} \exp\left( -\frac{(x_{i}-\mu)^{2}}{2\sigma^{2}} \right) \mathrm{d}\mu \mathrm{d}\sigma} \\ &= \frac{1}{\sqrt{n+1}}\left( 1 - \frac{\left( \frac{1}{n+1}{\sum}_{i=1}^{n} x_{i} \right)^{2}}{\frac{1}{n+1}{\sum}_{i=1}^{n} {x_{i}^{2}}} \right)^{-\frac{n}{2}} \end{array} $$

Formally, Jeffreys’ prior on *σ* is a ‘measure’ rather than a distribution, since it does not integrate to 1: clearly

7 $$ \begin{array}{@{}rcl@{}} {\int}_{0}^{\infty} {\mathbb P}_{\text{\textsc{j}}}(\sigma) \mathrm{d} \sigma = {\int}_{0}^{\infty} \frac{1}{\sigma} \mathrm{d} \sigma = \infty, \end{array} $$

Priors that integrate to infinity are often called *improper*. Use of such priors for nuisance parameters is not really a problem for Bayesian inference, since one can typically plug such priors into Bayes’ theorem anyway, and this leads to proper posteriors, i.e., posteriors that do integrate to one, and then the Bayesian machinery can go ahead. Since Jeffreys’ prior is meant to express that we have no clear prior knowledge about the variance, we would hope that Bayes would remain interpretable under optional stopping, no matter what the (unobservable) variance in our sampling distribution actually is. Remarkably, this is indeed the case: for all ${\sigma }_{0}^{2} > 0$, we have the following analogue of Eq. 6:

8 $$  \texttt{post-odds}( \mathcal{H}_{1}\/\text{vs.}\/\mathcal{H}_{0} | \sigma^{2}={\sigma}_{0}^{2},``\texttt{post-odds}(\mathcal{H}_{1}\/\text{vs.}\/\mathcal{H} | {x}_{1},\ldots,{x}_{\tau} =a") = a, $$

In words, this means that, given that the posterior odds (calculated based on Jeffreys’ prior, i.e., without knowing the variance) are equal to *a* *and* that the actual variance is ${\sigma ^{2}_{0}}$, the posterior odds are still *a*, irrespective of what ${\sigma ^{2}_{0}}$ actually is. This statement may be quite hard to interpret, so we proceed to illustrate it by simulation again.

**Fig. 2:**
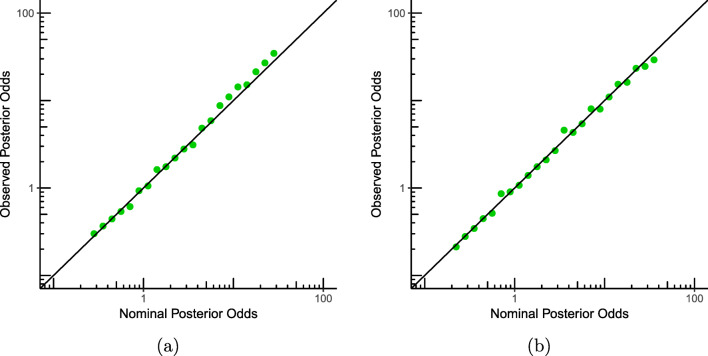
Calibration of the experiment of Section Example 1: Rouder’s example with a nuisance parameter, from 20,000 replicate experiments. **a** The observed posterior odds as a function of the nominal posterior odds. **b** The observed posterior odds as a function of the nominal posterior odds with optional stopping

To repeat Rouder’s experiment, we have to simulate data under both ${{\mathscr{H}}}_{0}$ and ${{\mathscr{H}}}_{1}$. To do this, we need to specify the variance *σ*^2^ of the normal distribution(s) from which we sample. Whereas, as in the previous experiment, we can sample the mean in ${{\mathscr{H}}}_{1}$ from the prior, for the variance we seem to run into a problem: it is not clear how one should sample from an improper prior. *𝜃*. But we cannot directly sample *σ* from an improper prior. As an alternative, we can pick any particular fixed *σ*^2^ to sample from, as we now illustrate. Let us first try *σ*^2^ = 1. Like Rouder’s example, we sample the mean of the alternative hypothesis ${\mathscr{H}}_{1}$ from the aforementioned normal distribution. Then, we sample ten data points from a normal distribution with the just sampled mean and the variance that we picked. For the null hypothesis ${\mathscr{H}}_{0}$ we sample the data from a normal distribution with mean zero and the same variance. We continue the experiment just as Rouder did: we calculate the posterior odds from 20,000 replicate experiments of ten generated observations for each hypothesis, and construct the histograms and the plot of the ratio of the counts to see if calibration is violated. In Fig. 2a we see the calibration plot for the experiment described above. In Fig. 2b we see the results for the same experiment, except that we performed optional stopping: we sampled until the posterior odds were at least 10-to-1 for ${\mathscr{H}}_{1}$, or the maximum of 25 observations was reached. We see that the posterior odds in this experiment with optional stopping are calibrated as well.

#### Prior calibration vs. strong calibration

Importantly, the same conclusion remains valid whether we sample data using *σ*^2^ = 1, or *σ*^2^ = 2, or any other value—in simulation terms Eq. 8 simply expresses that we get calibration (i.e., all points on the diagonal) no matter what *σ*^2^ we actually sample from: even though calculation of the posterior odds given a sample makes use of the prior ${\mathbb {P}}_{\text {\textsc {j}}}(\sigma ) = 1/\sigma $ and does not know the ‘true’ *σ*, calibration is retained under sampling under arbitrary ‘true’ *σ*. We say that the posterior odds are *prior-calibrated* for parameter *μ* and *strongly calibrated* for *σ*^2^. More generally and formally, consider general hypotheses ${\mathscr{H}}_{0}$ and ${\mathscr{H}}_{1}$ (not necessarily expressing that data are normal) that share parameters *γ*_0_,*γ*_1_ and suppose that Eq. 8 holds with *γ*_1_ in the role of *σ*^2^. Then we say that *γ*_0_ is prior-calibrated (to get calibration in simulations we need to draw it from the prior) and *γ*_1_ is strongly calibrated (calibration is obtained when drawing data under all possible *γ*_1_).

Notably, strong calibration is a special property of the chosen prior. if we had chosen another proper or improper prior to calculate the posterior odds (for example, the improper prior ${\mathbb {P}}^{\prime }(\sigma ) \propto \sigma ^{-2}$ has sometimes been used in this context) then the property that calibration under optional stopping is retained under any choice of *σ*^2^ will cease to hold; we will see examples below. The reason that ${\mathbb {P}}_{\text {\textsc {j}}}(\sigma ) \propto 1/\sigma $ has this nice property is that *σ* is a special type of nuisance parameter for which there exists a suitable group structure, relative to which both models are invariant (Eaton, 1989; Berger, Pericchi, & Varshavsky, 1998; Dass & Berger, 2003). This sounds more complicated than it is—in our example, the invariance is scale invariance: if we divide all outcomes by any fixed *σ* (multiply by 1/*σ*), then the Bayes factor remains unchanged; similarly, one may have for example location invariances.

If such group structure parameters are equipped with a special prior (which, for reasons to become clear, we shall term *type 0 prior*), then we obtain strong calibration, both for fixed sample sizes and under optional stopping, relative to these parameters.^3^ Jeffreys’ prior for the variance ${\mathbb {P}}_{\text {\textsc {j}}}(\sigma )$ is the type 0 prior for the variance nuisance parameter. (Dass and Berger, 2003) show that such priors can be defined for a large class of nuisance parameters—we will see the example of a prior on a common mean rather than a variance in Example 3 below; but there also exist cases with parameters that (at least intuitively) are nuisance parameters, for which type 0 priors do not exist; we give an example in the technical report (de Heide & Grünwald, 2018). For parameters of interest, including e.g., any parameter that does not occur in both models, type 0 priors never exist. Thus, strong calibration cannot be obtained for those parameters.

## When problems arise: Subjective vs. pragmatic and default priors

Bayesians view probabilities as degree of belief. The degree of belief an agent has before conducting the experiment, is expressed as a probability function. This *prior* is then updated with data from experiments, and the resulting *posterior* can be used to base decisions on.

For one pole of the spectrum of Bayesians, the pure *subjectivists*, this is the full story (de Finetti, 1937; Savage, 1972): any prior capturing the belief of the agent is allowed, but it should always be interpreted as the agent’s personal degree of belief; in Section Other conceptualizations of optional stopping we explain what such a ‘belief’ really means.

On the other end of the spectrum, the *objective Bayesians* (Jeffreys, 1961; Berger, 2006) argue that degrees of belief should be restricted, ideally in such a way that they do not depend on the agent, and in the extreme case boil down to a single, rational, probability function, where a priori distributions represent indifference rather than subjective belief and a posteriori distributions represent ‘rational degrees of confirmation’ rather than subjective belief. Ideally, in any given situation there should then just be a single appropriate prior. Most objective Bayesians do not take such an extreme stance, recommending instead *default* priors to be used whenever only very little a priori knowledge is available. These make a *default* choice for the functional form of a distribution (e.g., Cauchy) but often have one or two parameters that can be specified in a subjective way. These may then be replaced by more informative priors when more knowledge becomes available after all. We will see several examples of such default priors below.

So what category of priors is used in practice? Recent papers that advocate the use of Bayesian methods within psychology such as Rouder et al., (2009), Rouder et al., (2012), and Jamil et al., (2016) are mostly based on default priors. Within the statistics community, nowadays a pragmatic stance is by far the most common, in which priors are used that mix ‘default’ and ‘subjective’ aspects (Gelman, 2017) and that are also chosen to allow for computationally feasible inference. Very broadly speaking, we may say that there is a scale ranging from completely ‘objective’ (and hardly used) via ‘default’ (with a few, say 1 or 2 parameters to be filled in subjectively, i.e., based on prior knowledge) and ‘pragmatic’ (with functional forms of the prior based partly on prior knowledge, partly by defaults, and partly by convenience) to the fully subjective. Within the pragmatic stance, one explicitly acknowledges that one’s prior distribution may have some arbitrary aspects to it (e.g., chosen to make computations easier rather than reflecting true prior knowledge). It then becomes important to do sensitivity analyses: studying what happens if a modified prior is used or if data are sampled not by first sampling parameters *𝜃* from the prior and then data from ${\mathbb {P}}(\cdot \mid \theta )$ but rather directly from a fixed *𝜃* within a region that does not have overly small prior probability.^4^

The point of this article is that Rouder’s view on what constitutes ‘handling optional stopping’ is tailored towards a fully subjective interpretation of Bayes; as soon as one allows default and pragmatic priors, problems with optional stopping do occur (except for what we call type 0 priors). We can distinguish between three types of problems, depending on the type of prior that is used. We now give an overview of type of prior and problem, giving concrete examples later.

1. *Type 0 priors:* these are priors on parameters freely occurring in both hypotheses for which strong calibration (as with *σ*^2^ in Eq. 8) holds under optional stopping. This includes all right Haar priors on parameters that satisfy a group structure; Hendriksen et al., (2018) give a formal definition; (Dass and Berger, 2003; Berger et al., 1998) give an overview of such priors. We conjecture, but have no proof, that such right Haar priors on group structure parameters are the *only* priors allowing for strong calibration under optional stopping, i.e. the only type 0 Priors. Some, but not all so-called ‘nuisance parameters’ admit group structure/right Haar priors. For example, the variance in the *t* test setting does, but the mean in 2 × 2 contingency tables (de Heide & Grünwald, 2018) does not.
2. *Type I priors:* these are default or pragmatic priors that do *not* depend on any aspects of the experimental setup (such as the sample size) or the data (such as the values of covariates) and are not of type 0 above. Thus, strong calibration under optional stopping is violated with such priors—an example is the Cauchy prior in Example 2 of Section Example 2: Bayesian *t* test—The problem with type I priors*t* test with default priors below.
3. *Type II priors:* these are default and pragmatic priors that are not of type 0 or I: the priors may themselves depend on the experimental setup, such as the sample size, the covariates (design), or the stopping time itself, or other aspects of the data. Such priors are quite common in the Bayesian literature. Here, the problem is more serious: as we shall see, prior calibration is ill defined, and correspondingly Rouder’s experiments cannot be performed for such priors, and ‘handling optional stopping’ is in a sense impossible in principle. An example is the *g*-prior for regression as in Example 3 below or Jeffreys’ prior for the Bernoulli model as in Section Discrete data and type II priors below.

We illustrate the problems with type I and type II priors by further extending Rouder’s experiment to two extensions of our earlier setting, namely the Bayesian *t* test, going back to (Jeffreys, 1961) and advocated by (Rouder et al., 2009), and objective Bayesian linear regression, following (Liang, Paulo, Molina, Clyde, & Berger, 2008). Both methods are quite popular and use default Bayes factors based on default priors, to be used when no clear or very little prior knowledge is readily available.

### Example 2: Bayesian *t* test—The problem with type I priors

Suppose a researcher wants to test the effect of a new fertilizer on the growth of some wheat variety. The null hypothesis ${\mathscr{H}}_{0}$ states that there is no difference between the old and the new fertilizer, and the alternative hypothesis ${\mathscr{H}}_{1}$ states that the fertilizers have a different effect on the growth of the wheat. We assume that the length of the wheat is normally distributed with the same (unknown) variance under both fertilizers, and that with the old fertilizer, the mean is known to be *μ*_0_ = 1 meter. We now take a number of seeds and apply the new fertilizer to each of them. We let the wheat grow for a couple of weeks, and we measure the lengths. The null hypothesis ${\mathscr{H}}_{0}$ is thus: *μ* = *μ*_0_ = 1, and the alternative hypothesis ${\mathscr{H}}_{1}$ is that the mean of the group with the new fertilizer is different from 1 meter: *μ*≠ 1.

Again, we follow Rouder’s calibration check; again, the end goal is to illustrate a mathematical result, Eq. 9 below, which will be contrasted with Eq. 6. And again, to make the result concrete, we will first perform a simulation, generating data from both models and updating our prior beliefs from this data as before. We do this using the *Bayesian t test*, where Jeffreys’ prior ${\mathbb {P}}_{\text {\textsc {j}}}(\sigma )= 1/\sigma $ is placed on the standard deviation *σ* within both hypotheses ${\mathscr{H}}_{0}$ and ${\mathscr{H}}_{1}$. Within ${\mathscr{H}}_{0}$ we set the mean to *μ*_0_ = 1 and within ${\mathscr{H}}_{1}$, a standard Cauchy prior is placed on the effect size (*μ* − *μ*_0_)/*σ*; details are provided by Rouder et al., (2009). Once again, the nuisance parameter *σ* is equipped with an improper Jeffreys’ prior, so, like in Experiment 1 above and for the reasons detailed there, for simulating our data, we will choose a fixed value for *σ*; the experiments will give the same result regardless of the value we choose.

**Fig. 3:**
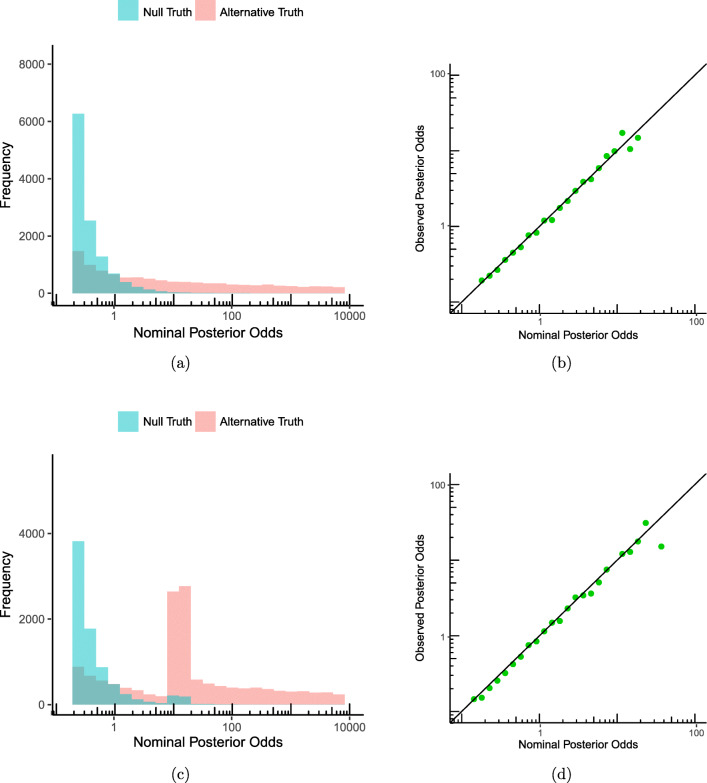
Calibration in the *t* test experiment, Section Example 2: Bayesian *t* test—The problem with type I priors, from 20,000 replicate experiments. **a** The distribution of posterior odds as a histogram under ${\mathscr{H}}_{0}$ and ${\mathscr{H}}_{1}$ in one figure. **b** The observed posterior odds as a function of the nominal posterior odds. **c** Distribution of the posterior odds with optional stopping. **d** The observed posterior odds as a function of the nominal posterior odds with optional stopping

We generate ten observations for each fertilizer under both models: for ${\mathscr{H}}_{0}$ we sample data from a normal distribution with mean *μ*_0_ = 1 meter and we pick the variance *σ*^2^ = 1. For ${\mathscr{H}}_{1}$ we sample data from a normal distribution where the variance is 1 as well, and the mean is determined by the effect size above. We adopt a Cauchy prior to express our beliefs about what values of the effect size are likely, which is mathematically equivalent to the effect size being sampled from a standard Cauchy distribution. We follow Rouder’s experiment further, and set our prior odds on ${\mathscr{H}}_{0}$ and ${\mathscr{H}}_{1}$, before observing the data, to 1-to-1. We sample ten data points from each of the hypotheses, and we calculate the Bayes factors. We repeat this procedure 20,000 times. Then, we bin the 20,000 resulting Bayes factors and construct a histogram. In Fig. 3a, we see the distribution of the posterior odds when either the null or the alternative are true in one figure. In Fig. 3b we see the calibration plot for this data from which Rouder checks the interpretation of the posterior odds: the observed posterior odds is the ratio of the two histograms, where the width of the bins is 0.1 on the log scale. The posterior odds are calibrated, in accordance with Rouder’s experiments. We repeated the experiment with the difference that in each of the 40,000 experiments we sampled more data points until the posterior odds were at least 10-to-1, or the maximum number of 25 data points was reached. The histograms for this experiment are in Fig. 3c. In Fig. 3d we can see that, as expected, the posterior odds are calibrated under optional stopping as well.

Since *σ*^2^ is a nuisance parameter equipped with its type 0 prior, it does not matter what value we take when sampling data. We may ask ourselves what happens if, similarly, we fix particular values of the mean and sample from them, rather than from the prior; for sampling from ${\mathscr{H}}_{0}$, this does not change anything since the prior is concentrated on the single point *μ*_0_ = 1; in ${\mathscr{H}}_{1}$, this means we can basically pick any *μ* and sample from it. In other words, we will check whether we have strong calibration rather than prior-calibration not just for *σ*^2^, but also for the mean *μ*. We now first describe such an experiment, and will explain its importance further below.

**Fig. 4:**
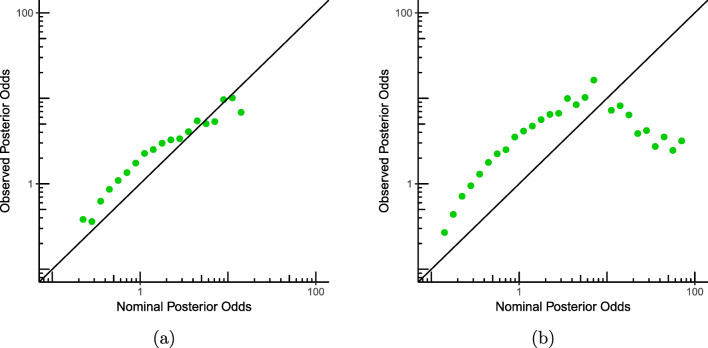
Calibration in the *t* test experiment with fixed values for the means of ${\mathscr{H}}_{0}$ and ${\mathscr{H}}_{1}$ (Section Example 2: Bayesian *t* test—The problem with type I priors, from 40,000 replicate experiments). **a** The observed posterior odds as a function of the nominal posterior odds. **b** The observed posterior odds as a function of the nominal posterior odds with optional stopping

We generate ten observations under both models. The mean length of the wheat is again set to be 1 m with the old fertilizer, and now we pick a particular value for the mean length of the wheat with the new fertilizer: 130 cm. For the variance, we again pick *σ*^2^ = 1. We continue to follow Rouder’s experiment and set our prior odds on ${\mathscr{H}}_{0}$ and ${\mathscr{H}}_{1}$, before observing the data, to 1-to-1. We sample 20,000 replicate experiments with 10 + 10 observations each, ten from one of the hypotheses (normal with mean 1 for ${\mathscr{H}}_{0}$) and ten from the other (normal with mean *μ* = 1.3 for ${\mathscr{H}}_{1}$), and we calculate the Bayes factors. In Fig. 4a, we see that calibration is, to some extent, violated: the points follow a line that is still approximately, but now not precisely, a straight line. Now what happens in this experiment under optional stopping? We repeated the experiment with the difference that we sampled more data points until the posterior odds were at least 10-to-1, or the maximum number of 25 data points was reached. In Fig. 4b, we see the results: calibration is now violated significantly—when we stop early the nominal posterior odds (on which our stopping rule was based) are on average significantly higher than the actual, observed posterior odds. We repeated the experiment with various choices of *μ*’s within ${\mathscr{H}}_{1}$, invariably getting similar results.^5^ In mathematical terms, this illustrates that when the stopping time *τ* is determined by optional stopping, then, for many *a* and $\mu ^{\prime }$,

9 $$ \begin{array}{@{}rcl@{}} \texttt{post-odds}(\mathcal{H}_{1}\/\text{vs.}\/{\mathcal{H}_{0}} | \mu = \mu^{\prime},``\texttt{post-odds}(\mathcal{H}_{1}\/\text{vs.}\/{\mathcal{H}_{0}} | x_{1},\ldots, x_{\tau})\\ = a") \text{ is very different from\ }\ a.\\ \end{array} $$

We conclude that strong calibration for the parameter of interest *μ* is violated somewhat for fixed sample sizes, but much more strongly under optional stopping. We did similar experiments for a different model with discrete data (see the technical report de Heide and Grünwald, 2018), once again getting the same result. We also did experiments in which the means of ${\mathscr{H}}_{1}$ were sampled from a different prior than the Cauchy: this also yielded plots which showed violation of calibration. Our experiments are all based on a one-sample *t* test; experiments with a two-sample *t* test and ANOVA (also with the same overall mean for both ${\mathscr{H}}_{0}$ and ${\mathscr{H}}_{1}$) yielded severe violation of strong calibration under optional stopping as well.

#### The issue

Why is this important? When checking Rouder’s prior-based calibration, we sampled the effect size from a Cauchy distribution, and then we sampled data from the realized effect size. We repeated this procedure many times to approximate the distribution on posterior odds by a histogram analogous to that in Fig. 1a. But do we really believe that such a histogram, based on the Cauchy prior, accurately reflects our beliefs about the data? The Cauchy prior was advocated by Jeffreys for the effect size corresponding to a location parameter *μ* because it has some desirable properties in hypothesis testing, i.e., when comparing two models (Ly, Verhagen, & Wagenmakers, 2016). For estimating a one-dimensional location parameter directly, Jeffreys (like most objective Bayesians) would advocate an improper uniform prior on *μ*. Thus, objective Bayesians may *change their prior depending on the inference task of interest*, even when they are dealing with data representing the same underlying phenomenon. It does then not seem realistic to study what happens if data are sampled from the prior; *the prior is used as a tool in inferring likely parameters or hypotheses, and not to be thought of as something that prescribes how actual data will arise or tend to look like*. This is the first reason why it is interesting to study not just prior calibration, but also strong calibration for the parameter of interest. One might object that the sampling from the prior done by Rouder, and us, was only done to illustrate the mathematical expression Eq. 6; perhaps sampling from the prior is not realistic but Eq. 6 is still meaningful? We think that, because of the mathematical equivalence, it does show that the relevance of Eq. 6 is questionable as soon as we use default priors.

Prior calibration in terms of Eq. 6—which indeed still holds^6^—*would* be meaningful if a Cauchy prior really described our prior beliefs about the data in the subjective Bayesian sense (explained in Section Other conceptualizations of optional stopping). But in this particular setup, the Cauchy distribution is highly unrealistic: it is a heavy tailed distribution, which means that the probability of getting very large values is not negligible, and it is very much higher than with, say, a Gaussian distribution. To make the intuition behind this concrete, say that we are interested in measuring the height of a type of corn that with the old fertilizer reaches on average 2 meter. The probability that a new fertilizer would have a mean effect of 6 m or more under a standard Cauchy distribution would be somewhat larger than 1 in 20. For comparison: under a standard Gaussian, this is as small as 9.87 ⋅ 10^− 10^. Do we really believe that it is quite probable (more than 1 in 20) that the fertilizer will enable the corn to grow to 8 m *on average*? Of course we could use a Cauchy with a different spread, but which one? Default Bayesians have emphasized that such choices should be made subjectively (i.e., based on informed prior guesses), but whatever value one choices, the chosen functional form of the prior (a Cauchy has, e.g., no variance) severely restricts the options, making any actual choice to some extent arbitrary. While growing crops (although a standard example in this context) may be particularly ill-suited to be modeled by heavy-tailed distributions, the same issue will arise with many other possible applications for the default Bayesian *t* test: one will be practically sure that the effect size will not exceed certain values (not too large, not too small, certainly not negative), but it may be very hard to specify exactly which values. As a purely objective Bayesian, this need not be such a big problem—one resorts to the default prior and uses it anyway; but one has to be aware that in that case, sampling from the prior—as done by Rouder—is not meaningful anymore, since the data one may get may be quite atypical for the underlying process one is modeling.

In practice, most Bayesians are pragmatic, striking a balance between ‘flat’, ‘uninformative’ priors, prior knowledge and ease of computation. In the present example, they might put a Gaussian prior with mean *μ* on the effect size instead, truncated at 0 to avoid negative means. But then there is the question what variance this Gaussian should have—as a pragmatic Bayesian, one has to acknowledge that there will always be arbitrary or ‘convenience’ aspects about one’s priors. This is the second reason why it is interesting to study not just prior calibration, but also strong calibration for the parameter of interest.

Thus, both from a purely objective and from a pragmatic Bayesian point of view, strong calibration is important. Except for nuisance parameters with type 0 priors, we cannot expect it to hold precisely (see (Gu, Hoijtink, & Mulder, 2016) for a related point)—but this is fine; like with any sensitivity or robustness test, we acknowledge that our prior is imperfect and we merely ask that our procedure remains reasonable, not perfect. And we see that by and large this is the case if we use a fixed sample size, but not if we perform optional stopping. In our view, this indicates that for pragmatic Bayesians using default priors, there is a real problem with optional stopping after all. However, within the taxonomy defined above, we implicitly used type I priors (Cauchy) here. Default priors are often of type II, and then, as we will see, the problems get significantly worse.

As a final note, we note that in our strong calibration experiment, we chose parameter values here which we deemed ‘reasonable’, by this we mean values which reside in a region of large prior density—i.e., we sampled from *μ* that are not too far from *μ*_0_. Sampling from *μ* in the tails of the prior would be akin to ‘really disbelieving our own prior’, and would be asking for trouble. We repeated the experiment for many other values of *μ* not too far from *μ*_0_ and always obtained similar results. Whether our choices of *μ* are truly reasonable is of course up to debate, but we feel that the burden of proof that our values are ‘unreasonable’ lies with those who want to show that Bayesian methods can deal with optional stopping even with default priors.

### Example 3: Bayesian linear regression and type II priors

We further extend the previous example to a setting of linear regression with fixed design. We employ the default Bayes factor for regression from the R package Bayesfactor (Morey & Rouder, 2015), based on Liang et al., 2008 and Zellner & Siow, 1980, see also Rouder & Morey, 2012. This function uses as default prior Jeffreys’ prior for the intercept *μ* and the variance (${\mathbb {P}}_{\text {\textsc {j}}}(\mu , \sigma ) \sim 1 / \sigma $), and a mixture of a normal and an inverse-gamma distribution for the regression coefficients, henceforth *g-prior*:

10 $$ \begin{array}{@{}rcl@{}} y &\sim& \text{N}\left( \mu + X\beta , \sigma^{2} \right), \\ \beta &\sim& \text{N}\left( 0, g \sigma^{2} n(X^{\prime}X)^{-1} \right), \\ g &\sim& \text{IG}\left( \frac{1}{2}, \frac{\sqrt{2}}{8} \right). \end{array} $$

Since the publication of Liang et al., (2008), this prior has become very popular as a default prior in Bayesian linear regression. Again, we provide an example concerning the growth of wheat. Suppose a researcher wants to investigate the relationship between the level of a fertilizer, and the growth of the crop. We can model this experiment by linear regression with fixed design. We add different levels of the fertilizer to pots with seeds: the first pot gets a dose of 0.1, the second 0.2, and so on up to the level 2. These are the *x*-values (covariates) of our simulation experiment. If we would like to repeat the examples of the previous sections and construct the calibration plots, we can generate the *y*-values—the increase or decrease in length of the wheat from the intercept *μ*—according to the proposed priors in Eq. 10. First we draw a *g* from an inverse gamma distribution, then we draw a *β* from the normal prior that we construct with the knowledge of the *x*-values, and we compute each *y*_*i*_ as the product of *β* and *x*_*i*_ plus Gaussian noise.

**Fig. 5:**
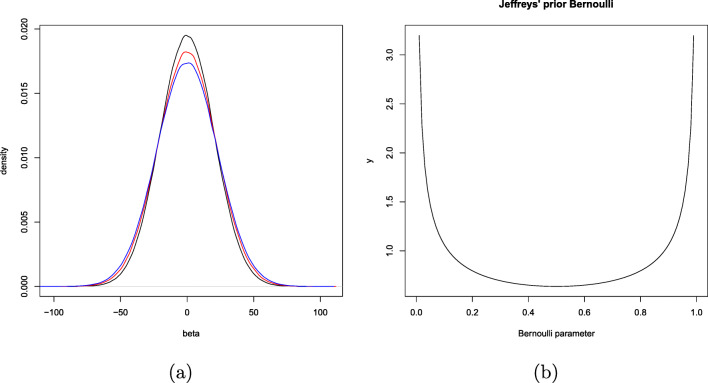
Default priors that depend on aspects of the experimental setup: **a***g*-priors for the regression example of Section Example 3: Bayesian linear regression and type II priors with different sample sizes: *n* = 20 (*black*), *n* = 23 (*red*), and *n* = 34 (*blue*). **b** Jeffreys’ prior for the Bernoulli model for the specific case that *n* is fixed in advance (no optional stopping): a beta (1/2,1/2) distribution

As we can see in Eq. 10, the prior on *β* contains a scaling factor that depends on the experimental setup—while it does not directly depend on the observations (*y*-values), it does depend on the design/covariates (*x*-values). If there is no optional stopping, then for a pragmatic Bayesian, the dependency on the *x*-values of the data is convenient to achieve appropriate scaling; it poses no real problems, since the whole model is conditional on *X*: the levels of fertilizer we administered to the plants. But under optional stopping, the dependency on *X* does become problematic, *for it is unclear which prior she should use!* If initially a design with 40 pots was planned (after each dose from 0.1 up to 2, another row of pots, one for each dose is added), but after adding three pots to the original twenty (so now we have two pots with the doses 0.1,0.2 and 0.3, and one with each other dose), the researcher decides to check whether the results already are interesting enough to stop, should she base her decision on the posterior reached with prior based the initially planned design with 40 pots, or the design at the moment of optional stopping with 23 pots? This is not clear, and it does make a difference, since the *g*-prior changes as more *x*-values become available. In Fig. 5a, we see three *g*-priors on the regression coefficient *β* for the same fixed value of *g*, the same *x*-values as described in the fertilizer experiment above, but increasing sample size. First, each dose is administered to one plant, yielding the black prior distribution for *β*. Next, three plants are added to the experiment, with doses 0.1,0.2 and 0.3, yielding the red distribution: wider and less peeked, and lastly, another 11 plants are added to the experiment, yielding the blue distribution which puts even less prior mass close to zero.

This problem may perhaps be pragmatically ‘solved’ in practice in two ways: either one could, as a rule, base the decision to stop at sample size *n* always using the prior for the given design at sample size *n*; or one could, as a rule, always use the design for the maximum sample size available. It is very unclear though whether there is any sense in which any of these two (or other) solutions ‘handle optional stopping’ convincingly. In the first case, the notion of prior calibration is ill defined, since $\text {post-odds}({\mathscr{H}}_{1}\/\text {vs.}\/{{\mathscr{H}}_{0}} | x_{1},\ldots , x_{\tau })$ in Eq. 6 is ill defined (if one tried to illustrate Eq. 6 by sampling, the procedure would be undefined since one would not know what prior to sample from until after one has stopped); in the second, one can perform it (by sampling *β* from the prior based on the design at the maximum sample size), but it seems rather meaningless, for if, for some reason or other, even more data were to become available later on, this would imply that the earlier sampled data were somehow ‘wrong’ and would have to be replaced.

What, then, about strong calibration? Fixing particular, ‘reasonable’ values of *β* is meaningful in this regression example. However (figures omitted), when we pick reasonable values for *β* instead of sampling *β* from the prior, we obtain again the conclusion that strong calibration is, on one hand, violated significantly under optional stopping (where the prior used in the decision to stop can be defined in either of the two ways defined above); but on the other hand, only violated mildly for fixed sample size settings. Using the taxonomy above, we conclude that optional stopping is a significant problem for Bayesians with type II priors.

### Discrete data and type II priors

Now let us turn to discrete data: we test whether a coin is fair or not. The data *D* consist of a sequence of *n*_1_ ones and *n*_0_ zeros. Under ${\mathcal H}_{0}$, the data are i.i.d. Bernoulli(1/2); under ${\mathcal H}_{1}$ they can be Bernoulli(*𝜃*) for any 0 ≤ *𝜃* ≤ 1 except 1/2, *𝜃* representing the bias of the coin. One standard objective and default Bayes method (in this case coinciding with an *MDL (minimum description length) method*, Grünwald, 2007) is to use Jeffreys’ prior for the Bernoulli model within ${\mathcal H}_{1}$. For fixed sample sizes, this prior is proper, and is given by

11 $$ {\mathbb{P}}_{\text{\textsc{j}}}(\theta) = \frac{1}{\sqrt{\theta(1- \theta)}} \cdot \frac{1}{\pi}, $$

where the factor 1/*π* is for normalization; see Fig. 5b. If we repeat Rouder’s experiment, and sample from this prior, then the probability that we would pick an extreme *𝜃*, within 0.01 of either 1 or 0, would be about ten times as large as the probability that we would pick a *𝜃* within the equally wide interval [0.49,0.51]. But, lacking real prior knowledge, do we really believe that such extreme values are much more probable than values around the middle? Most people would say we do not: under the subjective interpretation, i.e., if one really believes one’s prior in the common interpretation of ‘belief’ given in Section Other conceptualizations of optional stopping, then such a prior would imply a willingness to bet at certain stakes. Jeffreys’ prior is chosen in this case because it has desirable properties such as invariance under reparameterization and good frequentist properties, but not because it expresses any ‘real’ prior belief about some parameter values being more likely than others. This is reflected in the fact that in general, it depends on the stopping rule. Using the general definition of Jeffreys’ prior (see e.g., Berger, 1985), we see, for example, that in the Bernoulli model, if the sample size is not fixed in advance but depends on the data (for example, we stop sampling as soon as three consecutive 1s are observed), then, as a simple calculation shows, Jeffreys’ prior changes and even becomes improper (Jordan, 2010).

In the technical report (de Heide and Grünwald, 2018), we give another example of a common discrete setting, namely the 2 × 2 contingency table. Here the null hypothesis is a Bernoulli model and its parameter *𝜃* is intuitively a nuisance parameter, and thus strong calibration relative to this parameter would be especially desirable. However, the Bernoulli model does not admit a group structure, and hence neither Jeffreys’ nor any other prior we know of can serve as a type 0 prior, and strong calibration can presumably not be attained—the experiments show that it is certainly not attained if the default Gunel and Dickey Bayes factors (Jamil et al., 2016) are used (these are type II priors, so we need to be careful about what prior to use in the strong calibration experiment; see (de Heide & Grünwald, 2018) for details).

## Other conceptualizations of optional stopping

We have seen several problems with optional stopping under default and pragmatic priors. Yet it is known from the literature that, in some senses, optional stopping is indeed no problem for Bayesians (Lindley, 1957; Savage, 1972; Edwards et al., 1963; Good, 1991). What then, is shown in those papers? Interestingly, different authors show different things; we consider them in turn.

### Subjective Bayes optional stopping

The Bayesian pioneers (Lindley, 1957) and (Savage, 1972) consider a purely subjective Bayesian setting, appropriate if one truly believes one’s prior (and at first sight completely disconnected from strong calibration—but see the two quotations further below). But what does this mean? According to De Finetti, one of the two main founding fathers of modern, subjective Bayesian statistics, this implies a willingness to bet at small stakes, at the odds given by the prior.^7^ For example, a subjective Bayesian who would adopt Jeffreys’ prior ${\mathbb {P}}_{\text {\textsc {j}}}$ for the Bernoulli model as given by Eq. 11 would be willing to accept a gamble that pays off when the actual parameter lies close to the boundary, since the corresponding region has substantially higher probability, cf. the discussion underneath Eq. 11. For example, a gamble where one wins 11 cents if the actual Bernoulli parameter is in the set [0,0.01] ∪ [0.99,1] and pays 100 cents if it is in the set [0.49,0.51] and neither pays nor gains otherwise would be considered acceptable^8^ because this gamble has positive expected gain under ${\mathbb {P}}_{\text {\textsc {j}}}$. We asked several Bayesians who are willing to use Jeffreys’ prior for testing whether they would also be willing to accept such a gamble; most said no, indicating that they do not interpret Jeffreys prior the way a subjective Bayesian would.^9^

Now, if one adopts priors one really believes in in the above gambling sense, then Bayesian updating from prior to posterior is not affected by the employed stopping rule (Hendriksen et al., 2018); one ends up with the same posterior if one had decided the sample size *n* in advance or if it had been determined, for example, because one was satisfied with the results at this *n*. In this sense, a subjective Bayesian procedure does not depend on the stopping rule (as we have seen, this is certainly not the case in general for default Bayes procedures). This is the main point concerning optional stopping of (Lindley, 1957), also made by e.g., Savage, 1972; Bernardo & Smith, 1994, among many others. A second point made by (Lindley, 1957, p. 192) is that the decisions a Bayesian makes will “not, *on average*, be in error, when ignoring the stopping rule”. Here the “average” is really an expectation obtained by integrating *𝜃* over the prior, and then the data *D* over the distribution $\mathbb {P}(D \mid {\theta })$, making this claim very similar to prior calibration Eq. 6—once again, the claim is correct, but works only if one believes that sampling (or taking averages over) the prior gives rise to data of the type one would really expect; and if one would not be willing to bet based on the prior in the above sense, it indicates that perhaps one doesn’t really expect that data after all.

We cannot resist to add here that, while for a subjective Bayesian, prior-based calibration is sensible, even the founding fathers of subjective Bayes gave a warning against taking such a prior too seriously:^10^

“ Subjectivists should feel obligated to recognize that any opinion (so much more the initial one) is only vaguely acceptable... So it is important not only to know the exact answer for an exactly specified initial problem, *but what happens changing in a reasonable neighborhood the assumed initial opinion*” De Finetti, as quoted by Dempster, 1975.

— note that when we checked for strong calibration, we took parameter values *μ* which were not too unlikely under the prior, which one may perhaps view as ‘a reasonable neighborhood of the initial opinion’.

“ ...in practice the theory of personal probability is supposed to be an idealization of one’s own standard of behavior; the idealization is often imperfect in such a way that an aura of vagueness is attached to many judgments of personal probability...” (Savage, 1972).

Hence, one would expect that even a subjectivist would be interested in seeing what happens under a sensitivity analysis, for example checking for strong rather than prior-based calibration of the posterior. And even a subjectivist cannot escape the conclusion from our experiments that optional stopping leads to more brittle (more sensitive to the prior choice) inference than stopping at a fixed *n*.

### Frequentist optional stopping under ${\mathscr{H}}_{0}$

Interestingly, some other well-known Bayesian arguments claiming that ‘optional stopping is no problem for Bayesians’ really show that some Bayesian procedures can deal, in some cases, with optional stopping in a different, frequentist sense. These include (Edwards et al., 1963; Good, 1991) and many others (the difference between this justification and the above one by Lindley (1957) roughly corresponds to Example 1 vs. Example 2 in the appendix to Wagenmakers (2007)). We now explain this frequentist notion of optional stopping, emphasizing that some (but—contrary to what is claimed—by no means all!) tests advocated by Bayesians *do* handle optional stopping in this frequentist sense.

The (or at least, ‘a common’) frequentist interpretation of handling optional stopping is about controlling the type I error of an experiment. A type I error occurs when we reject the null hypothesis when it is true, also called a *false positive*. The probability of a type I error for a certain test is called the *significance level*, usually denoted by *α*, and in psychology the value of *α* is usually set to 0.05. A typical classical hypothesis test computes a test statistic from the data and uses it to calculate a *p* value. It rejects the null hypothesis if the *p* value is below the desired type I error level *α*. For other types of hypothesis tests, it is also a crucial property to control the type I error, by which we mean that we can make sure that the probability of making a type I error remains below our chosen significance level *α*. The frequentist interpretation of handling optional stopping is that the type I error guarantee holds if we do not determine the sampling plan—and thus the stopping rule—in advance, but we may stop when we see a significant result. As we know, see e.g., Wagenmakers, 2007, maintaining this guarantee under optional stopping is not possible with most classical *p* value-based hypothesis tests.

At first sight none of this seems applicable to Bayesian tests, which output posterior odds rather than a *p* value. However, in the case that ${\mathcal H}_{0}$ is *simple* (containing just one hypothesis, as in Example 0), there is a well-known intriguing connection between Bayes factors and type I error probabilities:

— if we reject *H*_0_ iff the posterior odds in favor of ${\mathscr{H}}_{0}$ are smaller than some fixed *α*, then we are guaranteed a type I error of at most *α*. And interestingly, this holds not just for fixed sample sizes but even under optional stopping. Thus, if one adopts the rejection rule above (reject iff the posterior odds are smaller than a fixed *α*), *for simple* ${\mathcal H}_{0}$*, frequentist optional stopping is no problem for Bayesians*. This is what was noted by Edwards et al., (1963) (using a different terminology) and Good (1991), based on what (Sanborn and Hills, 2014) call the *universal bound*, and what in probability theory is known as *Doob’s maximal inequality* (Doob, 1971); see also Vovk, Vereshchagin, Shen, and Shafer (2011) and van der Pas & Grünwald, 2018.

But what happens if ${\mathscr{H}}_{0}$ is composite? As was only shown very recently (Hendriksen et al., 2018), the Bayes factor still handles optional stopping in the frequentist sense if *all* free parameters in ${\mathscr{H}}_{0}$ are nuisance parameters observing a group structure and equipped with the corresponding type 0 prior and are shared with ${\mathscr{H}}_{1}$, an example being Jeffreys’ Bayesian *t* test of Section Example 2: Bayesian *t* test—The problem with type I priors. As explained by (Hendriksen et al., 2018), for general priors and composite ${\mathcal H}_{0}$ though, this is typically not the case; for example, the Gunel–Dickey default Bayes factors for2 × 2 tables (another composite ${\mathscr{H}}_{0}$) cannot handle optional stopping in the frequentist sense.

#### An empirical frequentist study of Bayesian optional stopping

(Schönbrodt, Wagenmakers, Zehetleitner, & Perugini, 2017) performed a thorough simulation study to analyze frequentist performance of optional stopping with Bayes factors both under ${\mathscr{H}}_{0}$ and under ${\mathscr{H}}_{1}$. They confined their analysis to the Bayesian *t* test, i.e., our Example 2, and found excellent results for the Bayesian optional stopping procedure *under a certain frequentist interpretation* of the Bayes factors (posterior odds). As to optional stopping under ${\mathscr{H}}_{0}$ (concerning type I error), this should not surprise us: in the Bayesian *t* test, all free parameters in ${\mathscr{H}}_{0}$ are equipped with type 0 priors, which, as we just stated, can handle optional stopping. We thus feel that one should be careful in extrapolating their results to other models such as those for contingency tables, which do not admit such priors. As to optional stopping under ${\mathscr{H}}_{1}$, the authors provide a table showing how, for any given effect size *δ* and desired level of type II error *β*, a threshold *B* can be determined such that the standard Bayesian *t* test with (essentially) the following optional stopping and decision rule, has type II error *β*:

Take at least 20 data points. After that, stop as soon as posterior odds are larger than *B* or smaller than 1/*B*; accept ${\mathscr{H}}_{0}$ if they are smaller than 1/*B*, and reject ${\mathscr{H}}_{0}$ if larger than *B*.

For example, if *δ* ≥ 0.3 and one takes *B* = 7 then the type II error will be smaller than 4*%* (see their Table 1). They also determined the average sample size needed before this procedure stops, and noted that this is considerably smaller than with the standard *t* test optimized for the given desired levels of type I and type II error and a priori expected effect size. Thus, if one determines the optional stopping threshold *B* in the Bayesian *t* test based on their table, one can use this Bayesian procedure as a frequentist testing method that significantly improves on the standard *t* test in terms of sample size. Under *this* frequentist interpretation (which relies on the specifics of a table), optional stopping with the *t* test is indeed unproblematic. Note that this does not contradict our findings in any way: our simulations show that if, when sampling, we fix an effect size in ${\mathscr{H}}_{1}$, then the posterior is biased under optional stopping, which means that we cannot interpret the posterior in a *Bayesian* way.

**Table 1:** Overview of several common default Bayes factors (from the R-package BayesFactor Morey and Rouder (2015)), and their robustness against different kinds of optional stopping (proofs can be found in Hendriksen et al., (2018))

	Prior Cal.	Strong Calibration	Freq. OS
Default Bayes Factors			
T test (Rouder et al., 2009)			
			
ANOVA (Rouder et al., 2012)			
			
Regression			
Rouder and Morey (2012)			
Contingency Tables			
Jamil et al., (2016)			
Bayes Factors with proper, fully		N/A	N/A
subjective priors (Rouder, 2014)			

‘Prior Cal.’ means ‘prior calibration’ and ‘Freq. OS’ means ‘frequentist optional stopping’. Between parentheses is the type of prior used (0, I, or II), in the taxonomy introduced in this paper. The indicates that, formally, prior calibration works for the priors, yet, because we are in the default setting, the Bayes factor is not fully subjective, so prior calibration is not too meaningful—which is just the main point of this paper

## Discussion and conclusion

When a researcher using Bayes factors for hypothesis testing truly believes in her prior, she can deal with optional stopping in the Bayesian senses just explained. However, these senses become problematic for every test that makes use of default priors, including all default Bayes factor tests advocated within the Bayesian Psychology community. Such ‘default’ or ‘objective’ priors cannot be interpreted in terms of willingness to bet, and sometimes (type II priors) depend on aspects of the problem at hand such as the stopping rule or the inference task of interest. To make sense of such priors generally, it thus seems necessary to *restrict* their use to their appropriate domain of reference—for example, Jeffreys’ prior for the Bernoulli model as given by Eq. 11 is okay for Bayes factor hypothesis testing with fixed sample size, but not for more complicated stopping rules. This idea, which is unfortunately almost totally lacking from the modern Bayesian literature, is the basis of a novel theory of the very concept of probability called *Safe Probability* which is being developed by one of us (Grünwald, 2013, 2018). That (mis)use of optional stopping is a serious problem in practice, is shown by, among others, John, Loewenstein, & Prelec, 2012; however, that paper is (implicitly) mostly about frequentist methods. It would be interesting to investigate to what extent optional stopping when combined with default Bayesian methods is actually a problem not just in theory but also in practice. This would, however, require substantial further study and simulation.

Rouder (2014) argues in response to (Sanborn and Hills, 2014) that the latter ‘evaluate and interpret Bayesian statistics as if they were frequentist statistics’, and that ‘the more germane question is whether Bayesian statistics are interpretable as *Bayesian statistics*’. Given the betting interpretation above, the essence here is that we need to make a distinction between the purely subjective and the pragmatic approach: we can certainly not evaluate and interpret *all* Bayesian statistics as *purely subjective* Bayesian statistics, what Rouder (2014) seems to imply. He advises Bayesians to use optional stopping—without any remark or restriction to purely subjective Bayesians, and for a readership of experimental psychologists who are in general not familiar with the different flavors of Bayesianism—as he writes further on: ‘Bayesians should consider optional stopping in practice. [...] Such an approach strikes me as justifiable and reasonable, perhaps with the caveat that such protocols be made explicit before data collection’. The crucial point here is that this can indeed be done when one works with a purely subjective Bayesian method, but not with the *default Bayes factors* developed for practical use in social science: both strong calibration and the frequentist type I error guarantees will typically be violated, and for Bayes factors involving type II priors, both prior and strong calibration are even undefined. In Table 1 we provide researchers with a simplified overview of four common default Bayes factors indicating which forms of optional stopping they can handle.

While some find the purely subjective Bayesian framework unsuitable for scientific research (see e.g., Berger, 2006), others deem it the only coherent approach to learning from data per se. We do not want to enter this discussion, and we do not have to, since in practice, nowadays most Bayesian statisticians tend to use priors which have both ‘default’ and ‘subjective’ aspects. Basically, one uses mathematically convenient priors (which one does not really believe, so they are not purely subjective—and hence, prior calibration is of limited relevance), but they are also chosen to be not overly unrealistic or to match, to some extent, prior knowledge one might have about a problem. This position is almost inevitable in Bayesian practice (especially since we would not like to burden practitioners with all the subtleties regarding objective and subjective Bayes), and we have no objections to it—but it does imply that, just like frequentists, Bayesians should be careful with optional stopping. For researchers who like to engage in optional stopping but care about frequentist concepts such as type I error and power, we recommend the *safe tests* of Grünwald, de Heide, & Koolen, 2019 based on the novel concept of *E-variables*: *E*-variables are related to, and sometimes coincide with, default Bayes factors, but tests based on *E*-variables invariably handle a variation of frequentist optional stopping. For example, the three default Bayes factors that handle frequentist optional stopping in Table 1 are also *E*-variables, but there exist other *E*-variables for these three settings that also handle optional stopping but achieve higher frequentist power; and there also exists an *E*-variable for contingency tables that, unlike the default Bayes factor, handles frequentist optional stopping.

## Footnotes

1 With some abuse of notation, we use $\mathbb {P}$ both to denote a generic probability distribution (defined on sets), and to denote its associated probability mass function and a probability density function (defined on elements of sets); whenever in this article we write $\mathbb {P}(z)$ where *z* takes values in a real-valued scalar or vector space, this should be read as *f*(*z*) where *f* is the density of $\mathbb {P}$.

2 The advantage of a normal is that it makes calculations relatively easy. A more common and perhaps more defensible choice is a Cauchy distribution, used in the ‘default Bayesian *t* test’, which we consider further below.

3 Technically, the type 0 prior for a given group structure is defined as the right Haar prior for the group (Berger et al., 1998): a unique (up to a constant) probability measure induced on the parameter space by the right Haar measure on the related group. Strong calibration is proven in general by Hendriksen et al., (2018) and Hendriksen (2017) for the special case of the 1-sample *t* test.

4 To witness, one of us recently spoke at the bi-annual OBAYES (Objective Bayes) conference, and noticed that a substantial fraction of the talks featured such fixed *𝜃*-analyses and/or used priors of type II below.

5 Invariably, strong calibration is violated both with and without optional stopping. In the experiments without optional stopping, the points still lie on an increasing and (approximately) straight line; the extent to which strong calibration is violated—the slope of the straight line—depends on the effect size. In the experiments with optional stopping, strong calibration is violated more strongly in the sense that the points do not follow a straight line anymore.

6 Note though that strong calibration still fails.

7 Savage, the other father, employs a slightly different conceptualization in terms of preference orderings over outcomes, but that need not concern us here.

8 One might object that actual Bernoulli parameters are never revealed and arguably do not exist; but one could replace the gamble by the following essentially equivalent gamble: a possibly biased coin is tossed 10,000 times, but rather than the full data only the average number of 1*s* will be revealed. If it is in the set [0, 0.01] ∪ [0.99, 1] one gains 11 cents and if it is in the set [0.49, 0.51] one pays 100 cents. If one really believes Jeffreys’ prior, this gamble would be considered acceptable.

9 Another example is the Cauchy prior with scale one on the standardized effect size (Rouder et al., 2012), as most would agree that this is not realistic in psychological research. Thanks to an anonymous reviewer for pointing this out.

10 Many thanks to Chris Holmes for bringing these quotations to our attention.
